# Supplementary material for: Social-ecological factors and preventive actions decrease the risk of dengue infection at the household-level: Results from a prospective dengue surveillance study in Machala, Ecuador
Source: PLoS Negl Trop Dis. 2017 Dec 18;11(12):e0006150. doi: 10.1371/journal.pntd.0006150 (PMC5771672; doi:10.1371/journal.pntd.0006150)
Supplement: S1 Text — (DOC) [file pntd.0006150.s002.doc]

**HOUSEHOLD SURVEY**

Subject Identification Number Date (dd/mm/yy):__________________

__ __ - __ __ __ __ __ - __ Name of interviewer: ________________________

**INFORMATION ABOUT THE HEAD OF THE HOUSEHOLD**

Age:_______ years Sex:  Male  Female

Does this person work?  Yes  No: Specify why not (e.g., retired, looking for work, disabled)_________________

If they do work, is this stable employment?  Yes  No

Do they earn the minimum wage?  Yes  No

What is their highest level of education?  None  Primary  Secondary  Post-secondary

**HOUSEHOLD DEMOGRAPHICS**

How many peole sleep in this household?_______ How many families sleep on this property?____________

Do you rent this house or do you own the home?  Rent  Own

Are there other families that rent on this property?  Yes  No

**ACCESS TO BASIC SERVICES AND WATER USE**

The water that this household receives is:

piped water inside the home  piped water outside the home  no piped water

When you open the faucet (either outside or inside the home) how often is there NO water?

there is always water  daily  2-3 times per week  other

What do you use to ventilate the household?  air conditioning  open the door/window  fan

Do you have a cistern or elevated water tank that is in use?  Yes  No

Do you store water apart from the water in the cistern or elevated water tank?  Yes always  Yes sometimes  No

IF YES, Why do you store water?  There is no piped water in my home

There are frequent interruptions in the water supply  It is convenient/habit  Other:_____________

For what do you use the stored water?  laundry  clean the house drinking water  cooking  bathing  to water the plants

Do you have animals?  dogs  cats  chickens/ducks  pigs  birds  other:_________

**PERCEPTIONS AND KNOWLEDGE OF DENGUE**

In your opinion, do you consider dengue to be a problem in your community?

Yes it is a serious problem  Yes it is a problem, but not so serious  No it’s not a problem

In your opinion, is dengue a severe, moderate or mild disease?

Severe  Moderate  Mild  Could be all three  I dont know

Dengue prevention in the household is:

easy  moderate  difficult  impossible – you can’t prevent dengue.

Have you ever received guidance on how to prevent dengue?  Yes  No  I don’t know

How is dengue transmitted?  mosquito  other:________________

Generally where do the mosquitoes reproduce that transmit dengue?

in containers, in water or clean water  other:________________

**PREVENTION ACTIONS**

What do you do to prevent dengue?

| screens on windows/doors | cut vegetation |
| --- | --- |
| apply repellent | apply chemicals to standing water |
| clean garbage | eliminate standing water |
| burn palosanto | pour burned diesel on the floors/puddles |
| cover water containers | fumigate in my house |
| shut windows/doors | use mosquito net |

If you apply a chemical to water to kill mosquito larvae, what do you use?

chlorine  Biolarvicida (BTI)  Abate/temefos  Otro:_________________

How often do you use it? _____ days

What difficulties do you have in taking these prevention actions?  no difficulties  lack of information

economic limitations  lack of time  too many mosquitoes  other:_______________

**CHARACTERISTICS OF THE HOUSEHOLD: OBSERVATIONS OF THE INVESTIGATOR**

1. The overall condition of the house is

Good (new, well maintained)  Regular  Bad (old, unpainted, uncared for)

2. The material of the external walls of the house?

brick of concrete blocks  wood  bamboo  other:_____________

3. How many bedrooms in the home? ______

4. Do doors and/or windows have screens?  Yes, all  Yes, some  No, none

5. What is the condition of the screens?  No screens  good (new)  normal  bad (old, holes)

6. Principal access to the household:  Paved street  dirt road  Other:__________

7. Are there abandoned properties or homes nearby?  yes  No

8. ¿Do you have a patio?  yes  No

9. Condition of the patio:

Very organized/clean (no garbage, garden well maintained)  Normal(little garbage)  Disorganized

10. Shade in the patio:  Sunny (<25% shaded)  Partial (25%-50%)  Shady (>50% shaded)
