# Supplementary material for: Social-ecological factors and preventive actions decrease the risk of dengue infection at the household-level: Results from a prospective dengue surveillance study in Machala, Ecuador
Source: PLoS Negl Trop Dis. 2017 Dec 18;11(12):e0006150. doi: 10.1371/journal.pntd.0006150 (PMC5771672; doi:10.1371/journal.pntd.0006150)
Supplement: S2 Text — (DOC) [file pntd.0006150.s003.doc]

**ENCUESTA DEL HOGAR**

Número de Identificación de la Casa Fecha (dd/mm/aa):__________________

__ __ - __ __ __ __ __ - __ Nombre del entrevistador: ________________________

**INFORMACION SOBRE EL/LA PRINCIPAL RESPONSABLE ECONÓMICO DEL MANTENIMIENTO DE SU FAMILIA**

Edad: _________ Años Género:  Masculino  Femenino

Se trabaja?  Sí  No: Especifica porque no (ej., jubilado, estudiante)_____________________________________

*Si se trabaja:* El trabajo es estable?  Sí  No

Esa persona gana el salario basico?  Sí  No

Cual es el nivel de educación mas alto al que esa persona asistio?

Ninguno  Primario  Secudario  Post-secundario

**EL HOGAR**

¿Cuántas personas en total duerman en su hogar?_______ ¿Cuántas familias duermen en esta propiedad?__________

¿Alquila su vivienda o es casa propia?  Alquilo  Casa propia

¿Hay otras familias que alquilen en esta vivienda?  Sí  No

**ACESO A SERVICIOS BASICOS Y USO DE AGUA**

El agua que recibe la vivienda es:

por tuberia dentro de la vivienda  por tuberia fuera de la vivienda  No recibe agua por tuberia

Cuando abres la llave (adentro o fuera de la casa) con que frequencia no cae el agua?

Siempre cae el agua  Interrupciones diarias o semanales  Otro:______________

El servicio higienico o escusado de la vivienda es por:  Alcantarillado  Pozo septico Otro:__________

¿Cada cuánto tiempo recogen la basura en su barrio?

Nunca  Diario  2-3 veces a la semana  Otro:________

¿Qué utiliza para ventilar/refrescar la casa?  Aire acondicionado  Abrir ventana/puerta  Ventilador

¿Tiene Usted cisterna o tanque elevado que esta en uso?  Sí  No

¿Almacena Usted agua aparte de en la cisterna o tanque elevada?  Si, todo el tiempo  Si, a veces  No

SI ALMACENA AGUA: ¿Porqué almacena agua (*lee las opciones y marque todos las que apliquen*)?

No hay agua entubada adentro de la casa  El agua se corta con frequencia

Es conveniente/hábito  Otro:_____________

¿Para qué se utiliza el agua almacenado (*lee las opciones*)?  Para lavar  Para limpiar la casa  Para tomar (consumo humano)  Para concinar  Para bañar  Para regar a las plantas

Tienen animales (*lee las opciones*)?  perros  gatos  pollos/patos  chanchos  pájaros  Otros:_________

**PERCEPCIONES Y CONOCIMIENTO SOBRE DENGUE**

En su opinión, ¿considera que el dengue es un problema en su comunidad? (*Lea todas las opciones*)

Si, es un problema muy serio  Si, es un problema, pero no tan serio  No es un problema

En su opinión, ¿el dengue es una enfermedad leve, moderada, o grave?

Grave  Moderada Leve  Podria ser las tres  No sabe

La prevencion de dengue en el hogar es (*Lea todas las opciones*):

facil  moderada  dificil  imposible (no hay como prevenir el dengue)

¿Alguna vez ha recibido sugerencias de cómo prevenir y/o controlar el dengue?  Si  No  No sabe

¿Cómo se transmite el dengue de una persona a otra? (no lea las opciones):

mosquito/mosco  otro:________________

¿Generalmente, en dónde se crían los mosquitos que transmiten el dengue? (no lea las opciones):

en recipientes, en agua o agua limpia  otro:________________

**MEDIDAS DE PREVENCION**

¿Qué hace usted para prevenir el dengue? (No lee las opciones. Marque tódas las que apliquen):

| Usar mallas en ventanas y puertas | Cortar el monte (plantas) |
| --- | --- |
| Aplicarme repelente. | Agrega quimicos en el agua para matar la larva |
| Limpieza de basura. | Eliminar agua almacenada. |
| Quemar Palosanto o rollos chinos. | Regar diesel quemado en los pozos o en el piso |
| Tapar depositos de agua. | Fumigación (rociamiento) al interior de la casa |
| Cerrar ventanas y puertas | Usar toldo/malla contra mosquitos |

Si usted agrega algun quimico en el agua para matar las larvas de mosquitos, ¿cuál se utiliza?

Cloro  Biolarvicida  Abate  Otro:_________________

¿Cada cuantos dias lo utiliza? _____ dias

¿Cuáles son las dificultades que tiene Ud. para tomar estas medidas de prevencion? (*Lea todas las opciones y marque todos los que apliquen*):  No hay dificultades  falta de informacion  falta economico  falta de tiempo

hay demasiado mosquitos  Otros:_______________

**CARACTERISTICAS DE LA VIVIENDA: OBSERVACIONES DEL ENCUESTADOR**

1. El estado en general de la casa es:

Bueno (nueva, bien mantenida)  Regular  Malo (viejo, despintado, descuidado)

2. El material predominante de las paredes exteriores de la vivienda?

ladrillo o bloque  madera  caña revestida o bahareque  Otro:_____________

3. ¿Cuántas habitaciones para dormir tiene la vivienda? ______

4. ¿Las ventanas y/o puertas tienen mallas?  Sí, todas  Si, algunas  No, ninguna

5. ¿Cuál es el estado las mallas?  No hay mallas

Bueno (nueva, bien mantenida)  Regular  Malo (viejos, huecos)

6. Aceso principal a la vivienda:  Calle pavimentado  Calle de tierra  Otro:________________

7. ¿Hay viviendas deshabitadas o terrenos baldíos alrededor de la vivienda?  Sí  No

8. ¿Hay un patio trasero?  Sí  No

9. Condicion del patio:

Muy organizado/limpio (sin basura, jardin mantenido)  Media organizada (poca basura)  Desorganizada

10. Nivel de sombra en el patio:  Soleado (<25% sombra)  Parcial (25%-50%)  Sombreado (>50% sombra)
